# Using large language models for safety-related table summarization in clinical study reports

**DOI:** 10.1093/jamiaopen/ooae043

**Published:** 2024-05-29

**Authors:** Rogier Landman, Sean P Healey, Vittorio Loprinzo, Ulrike Kochendoerfer, Angela Russell Winnier, Peter V Henstock, Wenyi Lin, Aqiu Chen, Arthi Rajendran, Sushant Penshanwar, Sheraz Khan, Subha Madhavan

**Affiliations:** Pfizer Research and Development, New York, NY 10001, United States; Pfizer Research and Development, New York, NY 10001, United States; Pfizer Research and Development, New York, NY 10001, United States; Pfizer Research and Development, New York, NY 10001, United States; Pfizer Research and Development, New York, NY 10001, United States; Pfizer Research and Development, New York, NY 10001, United States; Pfizer Research and Development, New York, NY 10001, United States; Pfizer Research and Development, New York, NY 10001, United States; Pfizer Research and Development, New York, NY 10001, United States; Pfizer Research and Development, New York, NY 10001, United States; Pfizer Research and Development, New York, NY 10001, United States; Pfizer Research and Development, New York, NY 10001, United States

**Keywords:** generative artificial intelligence, natural language processing, large language models, GPT-3.5, regulatory documents, clinical trials, text summarization

## Abstract

**Objectives:**

The generation of structured documents for clinical trials is a promising application of large language models (LLMs). We share opportunities, insights, and challenges from a competitive challenge that used LLMs for automating clinical trial documentation.

**Materials and Methods:**

As part of a challenge initiated by Pfizer (organizer), several teams (participant) created a pilot for generating summaries of safety tables for clinical study reports (CSRs). Our evaluation framework used automated metrics and expert reviews to assess the quality of AI-generated documents.

**Results:**

The comparative analysis revealed differences in performance across solutions, particularly in factual accuracy and lean writing. Most participants employed prompt engineering with generative pre-trained transformer (GPT) models.

**Discussion:**

We discuss areas for improvement, including better ingestion of tables, addition of context and fine-tuning.

**Conclusion:**

The challenge results demonstrate the potential of LLMs in automating table summarization in CSRs while also revealing the importance of human involvement and continued research to optimize this technology.

## Background and significance

The clinical study report (CSR) is a highly structured document that follows the format outlined in ICH E3 CSR.^1^^,^^2^ One time-intensive aspect of preparing a CSR is the review and description of safety data.^3^

LLMs are artificial neural networks^4^ which achieve text generation capabilities by leveraging massive amounts of data to learn billions of parameters during training.^5^^,^^6^ They are believed to acquire knowledge regarding syntax, semantics, and underlying “ontology” of human language.^7^^,^^8^ The success of ChatGPT passing the United States Medical Licensing Exam (USMLE)^9–11^ signals a potential breakthrough in LLMs’ ability to generate clinical insights.

One challenging aspect of automating CSR creation is extracting relevant information from tables. Clinical meaningfulness holds utmost importance. Achieving it often necessitates inclusion of supplementary information, such as comprehensive clinical expertise, and the study protocol. Furthermore, inference could emerge through connections across tables.^12^

There currently exists no software for CSR generation that uses LLMs as the main engine. A challenge was organized to examine what can be achieved using this technology. Participants were blinded to each other’s solution to foster independence and to apply their unique capabilities without bias. To define scope appropriate for a 6-week challenge,^13^ this experiment focused on the CSR Safety Summary section only, for a single therapeutic area, namely Inflammation and Immunology. Participants were challenged with generation of summary text for the sub-sections on Adverse Events, Deaths, Laboratory Results, Vital Signs, Electrocardiograms and Physical Examination Findings.

## Methods

The challenge was conducted between August 16 and October 5, 2023, with the initial call for submissions receiving a positive response from 23 external business entities from the United States, India, Germany, Ireland, France, Israel, United Kingdom, and Czech Republic. Based on these initial written proposals, six entities (technology companies of varied size) were selected to participate in the challenge. The text of the challenge statement can be found in Supplementary materials. Participants were not compensated but competed for the opportunity to collaborate with the organizer in the future.

Safety outputs of 72 CSRs from recently completed studies were identified for the training and test sets. These data are highly representative of what is currently being used by clinical small and medium-sized enterprises (SMEs) to prepare a CSR. Tables were supplied in the exact format that is currently used (HTML for in-text tables, PDF for out-of-text tables). The training set included studies from phase 1 to 3 trials; 58% of studies from phase 1, and 42% phases 2 and 3. In total, it included CSRs from 17 different drug assets covering a wide variety of safety-related events.

The CSRs were divided into 70% model training and the remaining 30% reserved for testing purposes. Training data included the CSR body text, safety summary data tables, protocols, and the safety narrative plans. Testing data included only the tables, protocols, and safety narrative plans. The task was to generate the text. No individual subject data were provided.

The models were developed by challenge participants over the course of 6 weeks using the training set and additional data provided by the organizer. Following this, the test set of tables (from 22 CSRs) was released to the participants, and they were required to produce the safety section of the CSRs within 24 h. The model output was evaluated by the organizer team, blinded to participant names.

### Environment and technical ground rules

The challenge was carried out by participant teams in a private, multi-tenancy compute workspace set up by the organizers on Databricks platform, utilizing a g5.24×large instance with four graphics processing units (GPUs). This environment provided personalized access and ensured data isolation within a shared infrastructure. Teams had access to GPT-3.5-turbo and lower versions. Fine-tuning was permitted on non-GPT, locally hosted models only. Vendors were evaluated according to three criteria domains: (1) *Technical score:* An evaluation to assess factual accuracy and text similarity scores via comparison to original CSRs. (2) *Business score:* An evaluation to assess overall usability of AI-generated text based on lean writing (concise, inferential, and relevant statements) and provenance (data traceability and extent of hallucination) for business users. (3) *Implementation score:* An evaluation to assess team’s presentations on the dimensions of technical approach, scalability, demo, and usability. Raters consisted of a multi-disciplinary team of 17 organizer members including data scientists, clinical statisticians, and medical writers (see challenge statement in Supplementary material).^6^

### Technical score

This included automated text evaluation scores and factual accuracy ratings. Automated metrics were text similarity scores comparing model output with original CSR text: Rouge-1 and Rouge-L.^14^^,^^15^ Numeric similarity was quantified by considering numeric values in the original CSR text and model output as two sets and calculating the Jaccard coefficient.^16^ Further, based on the original CSRs, the fraction of specific keywords (eg, unique safety issues within the study) present in the text was determined. Finally, semantic similarity was evaluated using GPT-4 in a fashion similar to GPT-score^17^ and G-Eval,^18^ by prompting GPT-4 to count the number of facts in the original CSR text (O) and counting how many facts in the model output text have the same semantic meaning as facts in the original text (M). The fraction M/O was used as a metric of semantic similarity. All scores were scaled to range 0-1. The mean of all automated text metrics was used for further analysis.

Factual accuracy ratings were performed manually by a team of raters. For each claim, factual accuracy was determined based on whether the claim is supported by the table data.^12^^,^^19^^,^^20^ All scores were scaled to range 0-1. The mean factual accuracy score across CSRs was used for further analysis. The mean of text metrics and factual accuracy constituted the technical score.

### Business score

This assessment was conducted by SMEs (organizer medical writing team) on the dimensions of Lean Writing and Provenance, which in turn consist of four and three items, respectively. The Lean Writing score evaluates inclusion of summary statements, presence of excessive repetitiveness, inclusion of inferential statements, and relevance of provided text. The Provenance score evaluates if sources are traceable, if sources came outside of provided data, and if there are any “Hallucinations” (claims that are not supported by the data provided).^21–23^ The scoring sheet can be found in Table S1, Supplementary material.

### Implementation

In addition to text outputs, participants gave presentations outlining their approach, as well as a demo. They described their plans, should they enter into collaboration with the organizer. Each presentation was rated on dimensions of Technical Approach, Scalability, Demo and Usability. For each of the dimensions, raters were given specific pointers to assess. See Supplementary Table S2 for details. The final score is the average of all scores after scaling to a range 0-1.

## Results

A key task in the CSR generation process is the ability to extract facts from study tables and listings and reformulate that information into concise, accurate text.^24^ Challenge participants approached this through diverse ingestion methods with varied success. Outputs often showed excellent comprehension of table structure, for example, distinguishing between treatment arms, although occasional parsing errors were observed.

**Figure 1.**
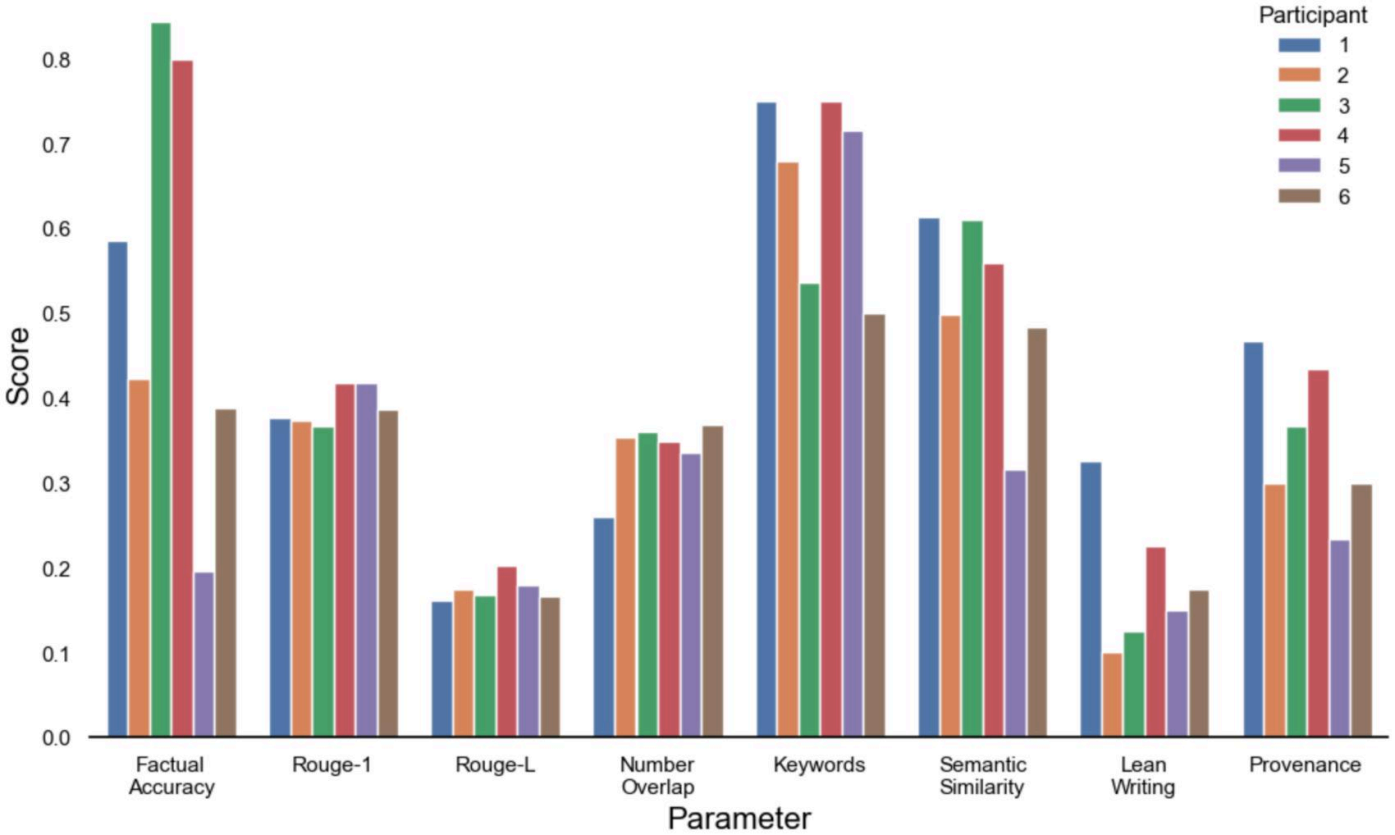
Scores in technical and business domain: (left to right) factual accuracy, automated similarity metrics Rouge-1, Rouge-L, number overlap, presence of keywords and semantic similarity, business domain scores lean writing and provenance.

The evaluation scores revealed differences in performance across the six teams (Figure 1). The teams diverged most in Factual Accuracy, indicating variability in precision of information prioritized for generation. Use of keywords and semantic similarity also varied widely, highlighting contrasts in utilizing relevant terms to inform study-specific safety issues and aligning content with standards. Similarly, teams’ scores demonstrated marked disparities in the domain-specific skills of Lean Writing and Provenance (see Supplementary material). On the other hand, metrics like Rouge-1, Rouge-L, and Number Overlap showed a narrower range of scores, pointing to a baseline competency shared by all teams in unigram matching, and sequence prediction. This stratification of results highlights the variability in different approaches employed by participants.

**Figure 2.**
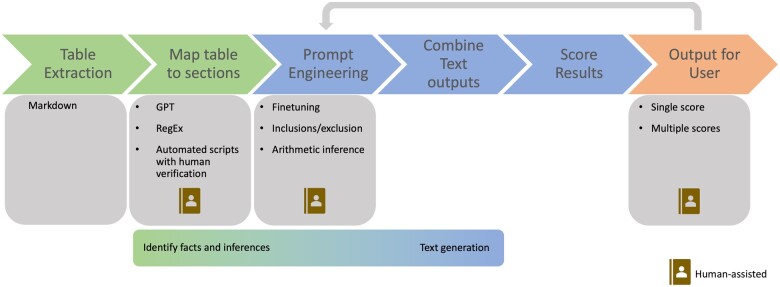
Approximate workflow used by most teams in the challenge.

Most teams in the challenge used the approach shown in Figure 2. To extract tables from CSRs, teams employed different approaches like GPT, regular expressions, and a combination of automated tools with human oversight to ensure precise data capture. Participants used a variety of strategies for the prompt engineering stage to enhance model performance—ranging from sophisticated filtering algorithms to the application of strict inclusion/exclusion criteria and the use of arithmetic logic to draw inferences (see Supplementary material for example prompts and solutions outline). Lastly, in the score results stage, we assessed the generated text outputs using a single score or a combination of metrics, addressing various aspects of summary quality. Variation was seen in the timing and level of involvement for a human expert in the loop. While some participants allowed humans to intervene at intermediate steps including table parsing (which led to much greater data extraction accuracy), others limited human feedback to prompt engineering only.

## Discussion

The challenge helped test generative AI and understand the opportunities and challenges with this technology for productivity improvement in pharma’s clinical development process. Central to this initiative, we developed a comprehensive evaluation framework for AI-generated output that employed a blend of automated metrics and SME reviews to assess document quality. This multifaceted approach not only ensured a robust validation, but also set a benchmark for scoring that is adaptable to related applications.

One limitation of the current study is the GPT version that was used, and the lack of fine-tuning capability for GPT models in our environment. The current Generative AI challenge environment was restricted to GPT-3.5-turbo and smaller non-GPT models. GPT-4,^25^ which became available late in the challenge, has a longer context, is more steerable using personas, and is less likely to fabricate facts.^26^^,^^27^ GPT4 will be tested in future work.

An important question at the outset of the challenge was whether it is better to use a finetuned model compared to GPT-3.5-Turbo which could not be finetuned at the time of the challenge.^28–30^ One team performed fine-tuning on a FLAN-T5-XL model,^31^ which showed higher ROUGE scores compared to prompt engineering. With more training data, this could be pursued in the future. Conversely, another team performed fine-tuning on a LLaMA 7B model,^32^ which gave a lift in quantitative metrics but showed hallucinations and erroneous summaries. Possible reasons include the small size of the training set,^33^ and the mix of one-to-one and many-to-many table to summary mappings.^34^ While fine-tuning GPT models show promise, large-scale deployment will require careful evaluation to ensure cost-effectiveness.

Aspects such as ranking of facts by importance, and inference cannot at present be automated, and therefore require SMEs.^35^ In evaluating model performance, factual accuracy should weigh higher than n-gram score comparisons to original CSR text. To enhance fact identification and inference, seven improvements are proposed: (1) Use of JSON instead of PDFs for accurate table ingestion; (2) Integration of protocols and safety narrative plans as contextual data; (3) Utilization of arithmetic modules for improved numerical processing; (4) Fine-tuning LLMs with additional CSRs; (5) Using reinforcement learning from human feedback (RLHF) to finetune models^33^^,^^36^^,^^37^; and (6) Using knowledge graphs for connecting relevant entities from protocols and tables.^38–41^

We expect productive gain from using LLMs of 20% time savings near-term and up to 50% after additional development and integration into business process. The impact will increase with inclusion of individual subject data. Broader use cases include additional CSR sections and therapeutic areas. Implementation of LLMs in production may encounter surmountable roadblocks such as the need for workflow adaptation, running LLMs in a secure environment, table standardization, added context and dealing with LLM system time-outs, for example, by using asynchronous LLM calls. Finally, proactive communication with regulators is essential to establish a clear understanding of the regulatory pathway.

In summary, this challenge demonstrated the potential for using large language models to automate safety-related table summarization in CSRs, while also highlighting areas for improvement. Key learnings include the need for human involvement, especially SMEs, to ensure accuracy and relevance. Continued research into integrating different AI methods with interactive human oversight will be important steps to realize the potential of this technology.

## Supplementary Material

ooae043_Supplementary_Data

## Data Availability

The data underlying this article will be shared on reasonable request to the corresponding author. See https://www.pfizer.com/science/clinical-trials/trial-data-and-results for more information.

## References

[1] Structure and content of clinical study reports, ICH E3, FDA; 1996. Accessed May 2024. https://www.fda.gov/media/71271/download

[2] Bhardwaj P , Sinha S, Yadav RK. Medical and scientific writing: time to go lean and mean. Perspect Clin Res. 2017;8(3):113-117. 28828305 10.4103/picr.PICR_11_17 PMC5543761

[3] Getz KA , Campo RA. New benchmarks characterizing growth in protocol design complexity. Ther Innov Regul Sci. 2018;52(1):22-28. 10.1177/2168479017713039 29714620

[4] Vaswani A , Shazeer N, Parmar N, et alAttention is all you need. Adv Neural Inf Process Syst. 2017;30. https://dl.acm.org/doi/10.5555/3295222.3295349

[5] Kojima T , Gu SS, Reid M, Matsuo Y, Iwasawa Y. Large language models are zero-shot reasoners. Adv Neural Inf Process Syst. 2022;35:22199-22213. https://proceedings.neurips.cc/paper_files/paper/2022/hash/8bb0d291acd4acf06ef112099c16f326-Abstract-Conference.html

[6] Brown T , Mann B, Ryder N, et alLanguage models are few-shot learners. Adv Neural Inf Process Syst. 2020;33:1877-1901. https://papers.nips.cc/paper/2020/hash/1457c0d6bfcb4967418bfb8ac142f64a-Abstract.html

[7] Wei J , Tay Y, Bommasani R, et al Emergent abilities of large language models. *Trans Mach Learn Res*. 2022. https://openreview.net/forum?id=CfzIsWWBlo

[8] Webb T , Holyoak KJ, Lu H. Emergent analogical reasoning in large language models. *Nat Hum Behav.* 2023;7(9):1526-1541. 10.1038/s41562-023-01659-w 37524930

[9] Kung TH , Cheatham M, Medenilla A, et al Performance of ChatGPT on USMLE: potential for AI-assisted medical education using large language models. *PLOS Digit Health*. 2023;2(2):e0000198. 10.1371/journal.pdig.0000198 PMC9931230 36812645

[10] Gilson A , Safranek CW, Huang T, et al How does ChatGPT perform on the United States medical licensing examination? The implications of large language models for medical education and knowledge assessment. JMIR Med Educ. 2023;9(1):e45312. 10.2196/45312 36753318 PMC9947764

[11] Nori H , King N, McKinney SM, Carignan D, Horvitz E. Capabilities of GPT-4 on medical challenge problems. *arXiv*, arXiv:230313375, 2023, preprint: not peer reviewed. 10.48550/arXiv.2303.13375

[12] Deng Y , Rosenberg D, Mann G. Challenges in end-to-end neural scientific table recognition. In: *2019 International Conference on Document Analysis and Recognition (ICDAR)*. IEEE; 2019:894-901. 10.1109/ICDAR.2019.00148

[13] Pfizer's Breakthrough Change Accelerator: CSR.Gen. Accessed May 2024. https://www.breakthroughchangeaccelerator.com/csrgen

[14] Lin C-Y. Rouge: a package for automatic evaluation of summaries. In: *Proceedings of Workshop on Text Summarization of ACL*. Barcelona, Spain: Association for Computational Linguistics; 2004:74-81. https://aclanthology.org/W04-1013

[15] Gomaa WH , Fahmy AA. A survey of text similarity approaches. Int J Comp Appl. 2013;68(13):13-18.

[16] Huang A. Similarity measures for text document clustering. In: *Proceedings of the sixth New Zealand computer science research student conference**(NZCSRSC2008)*, Christchurch, New Zealand; 2008.

[17] Fu J , Ng S-K, Jiang Z, Liu P. GPTscore: evaluate as you desire. *arXiv*, arXiv:230204166, 2023, preprint: not peer reviewed. 10.48550/arXiv.2302.04166

[18] Liu Y , Iter D, Xu Y, Wang S, Xu R, Zhu C. G-Eval: NLG Evaluation using Gpt-4 with Better Human Alignment. In: *Proceedings of the 2023 Conference on Empirical Methods in Natural Language Processing.* Singapore: Association for Computational Linguistics; 2023:2511-2522. https://aclanthology.org/2023.emnlp-main.153/

[19] Gong H , Sun Y, Feng X, et al TableGPT: Few-shot Table-to-Text Generation with Table Structure Reconstruction and Content Matching. In: *Proceedings of the 28th International Conference on Computational Linguistics*. Barcelona, Spain (Online): International Committee on Computational Linguistics; 2020:1978-1988. https://aclanthology.org/2020.coling-main.179

[20] Douglas S , Hurst M, Quinn D. Using natural language processing for identifying and interpreting tables in plain text. In: *Proceedings of the Fourth Annual Symposium on Document Analysis and Information Retrieval*.1995:535-546.

[21] Guerreiro NM , Alves D, Waldendorf J, et al Hallucinations in large multilingual translation models. *Trans Assoc Comput Linguist*. 2023;11(1):1500-1517. 10.1162/tacl_a_00615

[22] Lee M. A mathematical investigation of hallucination and creativity in GPT models. *Mathematics*. 2023;11(10):2320.

[23] Azamfirei R , Kudchadkar SR, Fackler J. Large language models and the perils of their hallucinations. *Crit Care*. 2023;27(1):1-2. 10.1186/s13054-023-04393-x PMC10032023 36945051

[24] Johnson D , Goodman R, Patrinely J, et al Assessing the accuracy and reliability of AI-generated medical responses: an evaluation of the Chat-GPT model. Res Sq (Preprint). 2023. 10.21203/rs.3.rs-2566942/v1

[25] OpenAI. GPT-4 technical report. *arXiv* 2303.08774, 2023. preprint: not peer reviewed. 10.48550/arXiv.2303.08774

[26] Taloni A , Borselli M, Scarsi V, et al Comparative performance of humans versus GPT-4.0 and GPT-3.5 in the self-assessment program of American academy of ophthalmology. *Sci Rep*. 2023;13(1):18562. 10.1038/s41598-023-45837-2 PMC10613606 37899405

[27] Lin JC , Younessi DN, Kurapati SS, Tang OY, Scott IU. Comparison of GPT-3.5, GPT-4, and human user performance on a practice ophthalmology written examination. *Eye (Lond)*. 2023;37(17):3694-3695. 10.1038/s41433-023-02564-2 PMC10686407 37156862

[28] Fichtel L , Kalo J-C, Balke W-T. Prompt tuning or fine-tuning-investigating relational knowledge in pre-trained language models. In: *3rd Conference on Automated Knowledge Base Construction*. 2021 (Virtual). https://openreview.net/forum?id=o7sMlpr9yBW

[29] Gu Y , Tinn R, Cheng H, et al Domain-specific language model pretraining for biomedical natural language processing. ACM Trans Comput Healthc (HEALTH). 2021;3(1):1-23.

[30] Tinn R , Cheng H, Gu Y, et al Fine-tuning large neural language models for biomedical natural language processing. *Patterns*. 2023;4(4):100729. 10.1016/j.patter.2023.100729 PMC10140607 37123444

[31] Chung HW , Hou L, Longpre S, et al Scaling instruction-finetuned language models. *J Mach Learn Res*. 2024;25(70):1-53. https://www.jmlr.org/papers/volume25/23-0870/23-0870.pdf

[32] Touvron H , Martin L, Stone K, et al Llama 2: open foundation and fine-tuned chat models. *arXiv*, arXiv:230709288, 2023, preprint: not peer reviewed. 10.48550/arXiv.2307.09288

[33] Bakker M , Chadwick M, Sheahan H, et al Fine-tuning language models to find agreement among humans with diverse preferences. Adv Neural Inform Process Syst. 2022;35(1):38176-38189. https://openreview.net/forum?id=G5ADoRKiTyJ

[34] Wang Y , Zhang J, Zhai F, Xu J, Zong C. Three strategies to improve one-to-many multilingual translation. In: *Proceedings of the 2018 conference on empirical methods in natural language processing.* 2018:2955-2960.

[35] Chang Y , Wang X, Wang J, et al A survey on evaluation of large language models. *ACM Trans Intell Syst Technol.* 2023;15(3):1-45. 10.1145/3641289

[36] Peng B , Li C, He P, Galley M, Gao J. Instruction tuning with GPT-4. *arXiv*, arXiv:230403277, 2023, preprint: not peer reviewed. 10.48550/arXiv.2304.03277

[37] Casper S , Davies X, Shi C, et al Open problems and fundamental limitations of reinforcement learning from human feedback. *Transact Mach Learn Res.* 2023:2835-8856. https://openreview.net/forum?id=bx24KpJ4Eb

[38] Yasunaga M , Ren H, Bosselut A, Liang P, Leskovec J. QA-GNN: reasoning with language models and knowledge graphs for question answering. In: *Proceedings of the 2021 Conference of the North American Chapter of the Association for Computational Linguistics: Human Language Technologies*. Online. Association for Computational Linguistics; 2021:535-546. https://aclanthology.org/2021.naacl-main.45

[39] Fei H , Ren Y, Zhang Y, Ji D, Liang X. Enriching contextualized language model from knowledge graph for biomedical information extraction. *Brief Bioinform*. 2021;22(3):bbaa110. 10.1093/bib/bbaa110 32591802

[40] Zhu Y , Wang X, Chen J, et al LLMs for knowledge graph construction and reasoning: recent capabilities and future opportunities. *arXiv*, arXiv:230513168, 2023, preprint: not peer reviewed. 10.48550/arXiv.2305.13168

[41] Agarwal O , Ge H, Shakeri S, Al-Rfou R. Knowledge graph based synthetic corpus generation for knowledge-enhanced language model pre-training. In: *Proceedings of the 2021 Conference of the North American Chapter of the Association for Computational Linguistics: Human Language Technologies.* Online: Association for Computational Linguistics; 2020:3554-3565. 10.18653/v1/2021.naacl-main.278

